# Peroxymonosulfate activation by Fe–Mn Co-doped biochar for carbamazepine degradation

**DOI:** 10.1039/d3ra06065a

**Published:** 2024-01-02

**Authors:** Xinze He, Yunxia Luo, Yang Yi, Shuping Su, Wenzhen Qin

**Affiliations:** a School of Environmental and Chemical Engineering College, Nanchang Hangkong University Nanchang 330000 China nanuotong@163.com qwz1417@163.com; b Children's Hospital of Chongqing Medical University Chongqing 401122 China

## Abstract

Antibiotics in aquatic environments present a serious threat to the ecological environment and human health. Activation of carbon-catalyzed persulfate is a prospective approach for oxidizing antibiotics. There is a pressing need for inexpensive carbon catalysts of high quality. In this study, biochar (BC) modified by Fe, Mn and Fe@Mn was employed to activate peroxymonosulfate (PMS) to degrade carbamazepine (CBZ) in water. The surface of Fe@Mn BC had a dense, stalactite-like morphology comprising a square chassis that was elliptical. The catalyst Fe@Mn–BC possessed the optimal degradation effect (99%) on CBZ at 100 min. Electron paramagnetic resonance spectroscopy and the quenching spectrum suggested that ˙O_2_^−^ and ^1^O_2_ contributed to CBZ degradation.

## Abstract 2

Biochar (BC) modified by Fe@Mn was employed to activate PMS to degrade CBZ in water. The surface of Fe@Mn–BC presented a dense, stalactite-like morphology. The catalyst Fe@Mn–BC had an optimal degradation effect (99%) on CBZ at 100 min.

## Graphical abstract

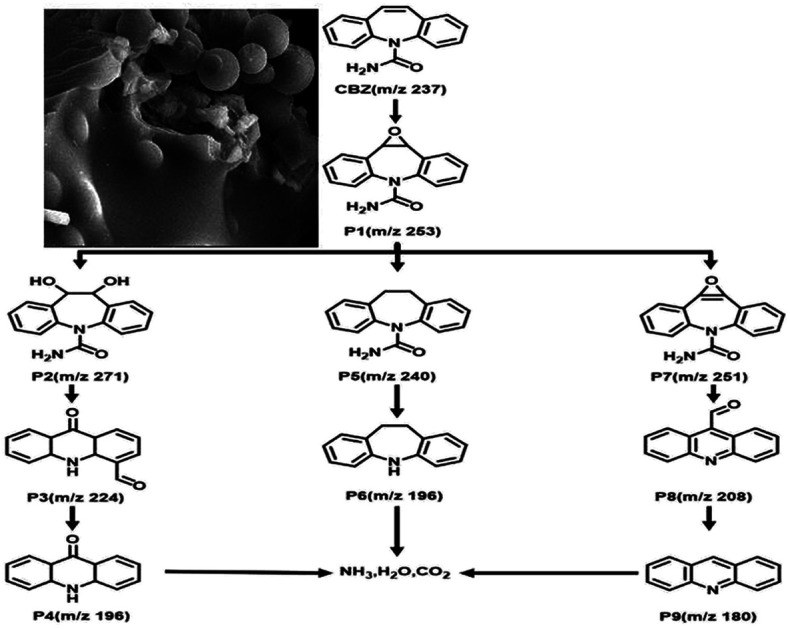

## 1. Introduction

Prolonged exposure to contamination by antibiotics can terrorize the security of hygrophilous ecosystems and constitute potential risks for human health.^1^ Carbamazepine (CBZ) is a typical medicinal product employed widely to treat diseases.^2,3^ However, with the extensive application of this compound, CBZ is released into the aquatic environment and causes adverse effects upon it. Therefore, developing a method for the efficient elimination of CBZ is a rational approach.^4–6^

In recent years, advanced oxidation process (AOPs) technology has drawn much attention due to its excellent effect on hard-to-degrade organic pollutants. Compared with traditional treatment methods, AOPs have a greater degradation capacity,^7,8^ which is considered to be an efficacious and prospective method for removing poisonous and durable organic materials. AOPs include methods involving peroxonosulfate (PMS),^9^ the Fenton reaction,^10,11^ electrochemistry,^12^ photocatalysis,^13^ ultrasonic irradiation,^14^ and ozone oxidation.^15^ Among these AOPs methods, PMS is considered as the best way to remove pollutants owing to its simple operation, strong oxidation capacity and application of a wide pH range.^16,17^ PMS can be activated by external factors (heat, UV radiation) and catalysts (*e.g.*, carbon-based materials and metal ions) to yield active species and gain efficient oxidation properties.^18^ PMS activation induced by electron transfer in carbon-based materials has attracted great interest in recent years.

Biochar (BC) is a carbon-based material. It is a byproduct of biomass pyrolysis, with highly dispersed reaction sites, rich pore structure, high surface area and adsorption ability.^19^ In addition, BC possesses rich functional groups (*e.g.*, carboxyl, phenol, hydroxyl, and acid anhydride) that promote contaminant removal.^20^ However, BC cannot provide sufficient electrons for PMS activation, which results in lower catalytic efficiency.^21^ Studies have shown that the introduction of metals can improve PMS activation.^22^ The difference in the standard redox potential of bimetallic materials can promote valence cycling and electron transfer.^23,24^ In addition, binary metal atomic clusters can be formed if two metals are doped onto BC, which increases the dispersion of active sites.^25^ Meanwhile, bimetallic doping decreases the infusion of metal ions, which also reduces the toxicity of the catalyst and makes the process more environmentally friendly.^26^ In particular, Fe and Mn are inexpensive and environmentally friendly.^27^ The generation of magnetic Fe oxides enhances the separability of catalysts.^28^ Simultaneously, the introduction of Fe and Mn can generate new active sites and greatly improve the conductivity of BC, which could facilitate electron transfer and, ultimately, increase PMS activation.

In the present study, BC was synthesised from soybean powder. Bimetallic Fe@Mn–BC catalysts with improved properties were prepared to overcome the agglomeration of metal ions and enable application in commercial products. The prepared Fe@Mn–BC catalysts were applied for PMS activation and CBZ degradation. Measurements were made using X-ray diffraction (XRD), X-ray photoelectron spectroscopy (XPS), and scanning electron microscopy (SEM).

## 2. Experimental

### 2.1 Synthesis of catalysts

Deionized (DI) water was employed to clean soybeans (Lianyungang Lianfeng Seed Industry, Jiangsu, China), which were then dried. A ball mill was used to mill the dried soybeans at 350 rpm for 6 h. Then, the products were calcined under a N_2_ atmosphere at 600 °C for 2 h in a tubular furnace. The materials were used as pristine soybean BC.

Fe and Mn co-doped BC were synthetized *via* a hydrothermal method. First, BC (2 g), KMnO_4_ (1.58 g; Macklin Biochemical Technology, Shanghai, China) and Fe powder (0.17 mol L^−1^; Hebei Lebo Metal Materials Technology, Hebei, China) were added to DI water (100 mL) to obtain a suspension A. Next, epichlorohydrin (10 mL) was dropped into suspension A to obtain suspension B. Then, suspension B was stirred for 2 h and heated in a rotatory shaker at 90 °C for 12 h. Then, the materials were centrifuged, washed and dried. The sample was termed Fe@Mn–BC. For comparison, Fe–BC and Mn–BC were obtained in the same way without KMnO_4_ or Ferrous powder, respectively.

### 2.2 Characterization

X-ray diffraction (Cu Kα; *λ* = 1.5406 Å; Bruker, Ettlingen, Germany) was employed to study the phase structure of materials. XPS using an Axis Ultra DLD system (Kratos Analytical, Manchester, UK) was applied to investigate the binding energy of elements. The surface compositions of materials were analyzed by Fourier transform infrared (FTIR) spectroscopy (Vertex 70; Bruker). SEM employing a Quanta 250 setup (FEI, Hillsboro, OR, USA) was used to study the surface morphology and microstructure. A three-electrode electrochemical workstation (CHI660D; CH Instruments, Bee Cave, TX, USA) was used to measure the photocurrent responses of materials. The surface area was measured by an analyzer (NOV 2000e; Quantachrome, Boynton Beach, FL, USA).

### 2.3 Catalytic properties

To determine the catalytic properties of the prepared products, degradation experiments of CBZ were studied in a conical flask. Briefly, samples (20 mg) were placed in a 100 mL solution of CBZ (10 mg L^−1^) and the mixture was stirred constantly. The adsorption equilibrium was achieved in the dark after 30 min. Then, (20 mg) was introduced in the solution stated above, and the degradation reaction started in the dark. The initial pH was adjusted to ∼7 by NaOH solution, and the degradation reaction last for 70 min in the dark. An aliquot (3 mL) was removed from the reaction mixture at regular intervals and centrifuged, and the supernatant was studied by a UV-vis spectrophotometer. Eqn (1) was selected to determine the degradation efficiency (*η*):1
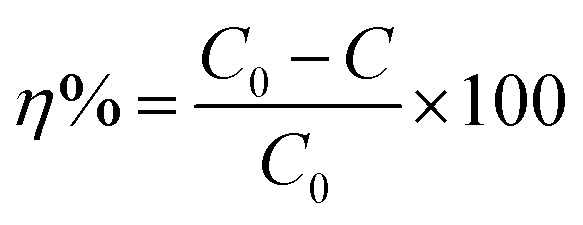
where *C*_0_ and *C* are the initial concentration and residual concentration of CBZ, respectively.

Meanwhile, the degradation efficiency under visible light was studied. Briefly, the CBZ mixture was exposed to a xenon lamp (300 W, 420 nm) instead of a dark condition. The influence on CBZ degradation of pH (3, 5, 7, 9 or 11) on different samples was studied. Furthermore, to ascertain the main reactive species degrading CBZ, quenching experiments were conducted. Furfuryl alcohol (FFA), *p*-benzoquinone (p-BQ) and *tert*-butyl alcohol (TBA) were employed as scavengers, all of which were from Macklin Biochemical Technology (Shanghai, China). In addition, the production of reactive free radicals was surveyed by electron paramagnetic resonance (EPR) spectroscopy, and the capture agents were DMPO and TEMP. When testing the reusability of catalysts, the sample was centrifuged (8000 rpm, 4 min) and dried for next degradation test, which was conducted on the third cycle.

## 3. Results and discussion

### 3.1 Morphology of samples

**Fig. 1:**
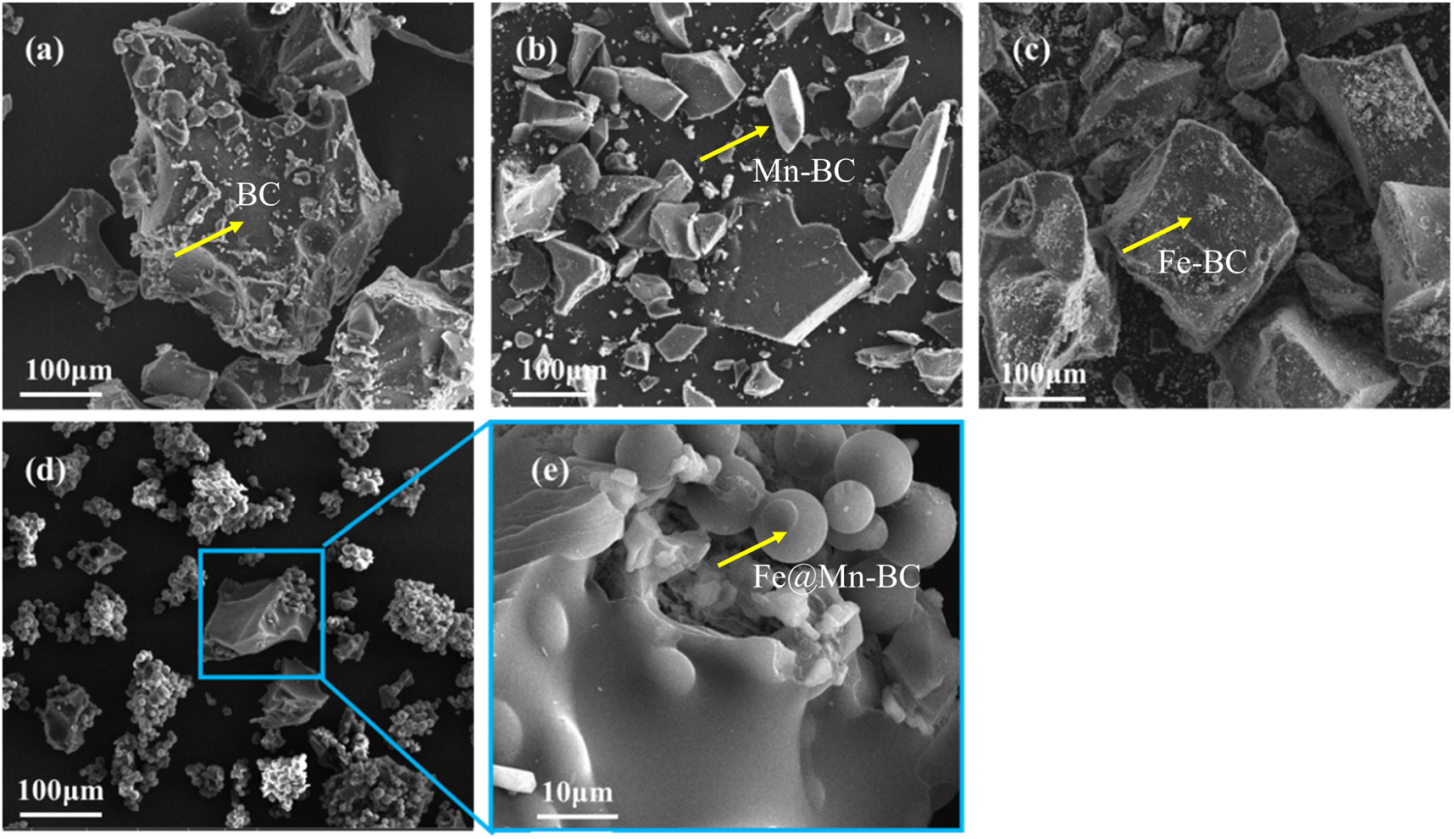
SEM images of (a) BC, (b) Mn–BC, (c) Fe–BC, and (d)–(e) Fe@Mn–BC.

**Fig. 2:**
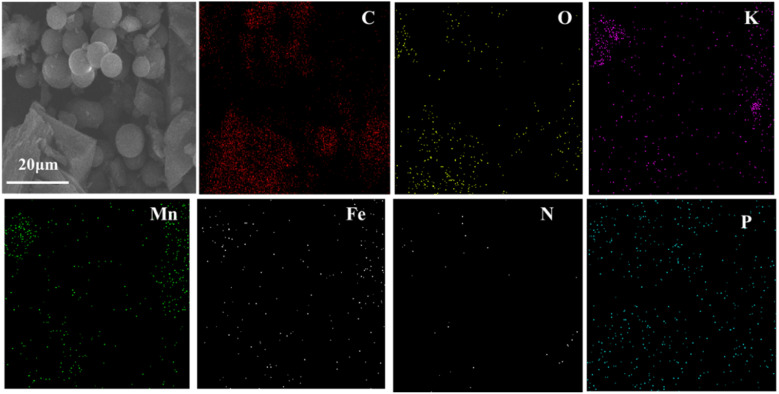
EDS images of Fe@Mn–BC.

SEM was applied to ascertain the morphology of samples. BC samples had an erratic block-like shape (Fig. 1a). After modification by Mn (Fig. 1b), a less erratic structure appeared in Mn–BC samples. Fig. 1c shows that many grains were deposited upon Fe–BC. In contrast, Fe@Mn–BC exhibited a dense, stalactite-like morphology with some spherical shapes (Fig. 1d and e), which were induced by the doping of Fe and Mn. Elemental mapping images revealed C, O, K, Mn, Fe, N and P elements in Fe@Mn–BC, and these elements were evenly dispersed (Fig. 2a–h), which suggested that Fe and Mn had been doped on the BC surface.

**Fig. 3:**
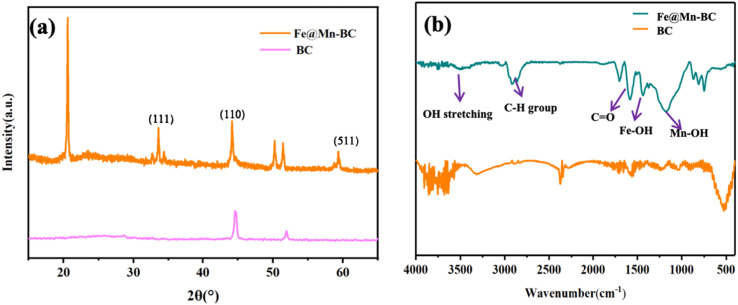
(a) XRD analysis and (b) FTIR spectroscopy of BC and Fe@Mn–BC.

XRD analysis of BC and Fe@Mn–BC samples is displayed in Fig. 3a. There was an obvious peak at 43.1 cm^−1^ in BC samples, which corresponded to the (100) plane of crystalline carbon.^29^ For Fe@Mn–BC, the diffraction peaks observed at 34.1 cm^−1^ corresponded to the (111) plane of MnO (PDF# 07-0230).^30^ The peaks at 43.5 and 59.4 cm^−1^ were ascribed to the (110) plane of Fe^0^ (JCPDS# 06-0696) and (511) plane of Fe_2_O_3_ (JCPDS# 39-1346), respectively,^31^ which confirmed the presence of Fe and MnO in Fe@Mn–BC samples. The chemical groups of BC and Fe@Mn–BC were studied by FTIR spectroscopy (Fig. 3b). Few function groups were observed in BC samples, and the peaks are weaker. However, Fe@Mn–BC had some absorption peaks, which indicated that Fe@Mn–BC had more diverse functional groups. The peak at 3430 cm^−1^ in Fe@Mn–BC was assigned to the OH stretching vibration.^32^ The characteristic peak at 2918 cm^−1^ belonged to the stretching vibration of the C–H group. The peaks at 1622 cm^−1^ corresponded to the binding vibration of C=O.^33^ The peaks at 1362 cm^−1^ and 1049 cm^−1^ were ascribed to the vibrations of Fe–OH and Mn–OH groups, respectively.^34^ The peaks at 697 cm^−1^ and 566 cm^−1^ can be classified as the functional groups of Mn–O and Fe_2_O_3_, respectively.^35,36^

**Fig. 4:**
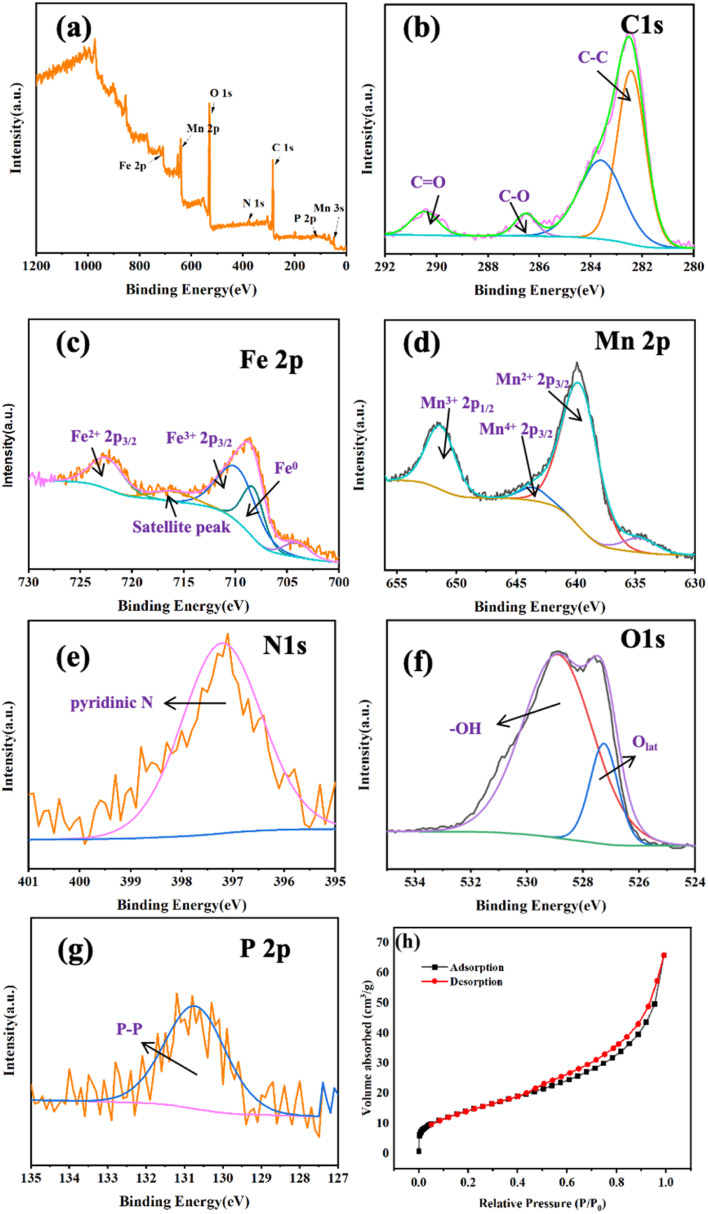
XPS spectra of Fe@Mn–BC (a). The peaks for C 1s (b), Fe 2p (c), Mn 2p (d), N 1s (e), O 1s (f) and P 2p (g), and BET properties of Fe@Mn–BC (h).

The chemical elements on the surface of Fe@Mn–BC were investigated further by XPS. The C 1s, Fe 2p, Mn 2p, N 1s, O 1s and P 2p peaks were observed in the FTIR spectra for Fe@Mn–BC (Fig. 4a). The C 1s peak (Fig. 4b) was split into three peaks, which are corresponded to the C–C bond (282.1 eV), C–O bond (286.1 eV) and C=O bond (290.8 eV).^37^ The Fe 2p spectrum is displayed in Fig. 4c. The peak at 709.4 eV was ascribed to Fe^0^ 2p_1/2_.^38^ The peaks at 713 eV corresponded to 2p_3/2_ of Fe^3+^, respectively. The peak at 723 eV belonged to Fe^2+^ 2p_3/2_.^39,40^Fig. 4d shows the XPS spectra of Mn 2p. The peaks at 639.6 eV and 644.7 eV represent Mn 2p_3/2_, whereas the peak at 651.5 eV is Mn 2p_1/2_, thereby indicating the existence of Mn^2+^, Mn^3+^ and Mn^4+^.^41,42^ The deconvolutions of N 1s were assigned to pyridinic N (397.7 eV), as shown in Fig. 4e.^43^Fig. 4f shows the XPS spectrum of O 1s, which was split into two peaks. The peak at 527.3 eV was generated by lattice oxygen in the Fe@Mn–BC phase, and the peak at 529.7 eV was attributed to O–H.^44^ For the P 2p spectrum (Fig. 4g), the peak at 130.6 eV corresponded to P–P. Fig. 4h shows the isotherm of Fe@Mn–BC. Based on these results, Fe and Mn were doped on BC. It is apparent from Fig. 4h that Fe@Mn–BC exhibited a type-IV isotherm and a H1-type loop, in line with the IUPAC classification.^45^ These observations indicate that Fe@Mn–BC possessed a mesoporous structure.^46^ The specific surface area of BC was increased by doping Mn and Fe, providing more active sites on the surface and enhancing the adsorption capacity of reactants.

### 3.2 Analysis of reactive oxygen species (ROS) in the catalytic process

**Fig. 5:**
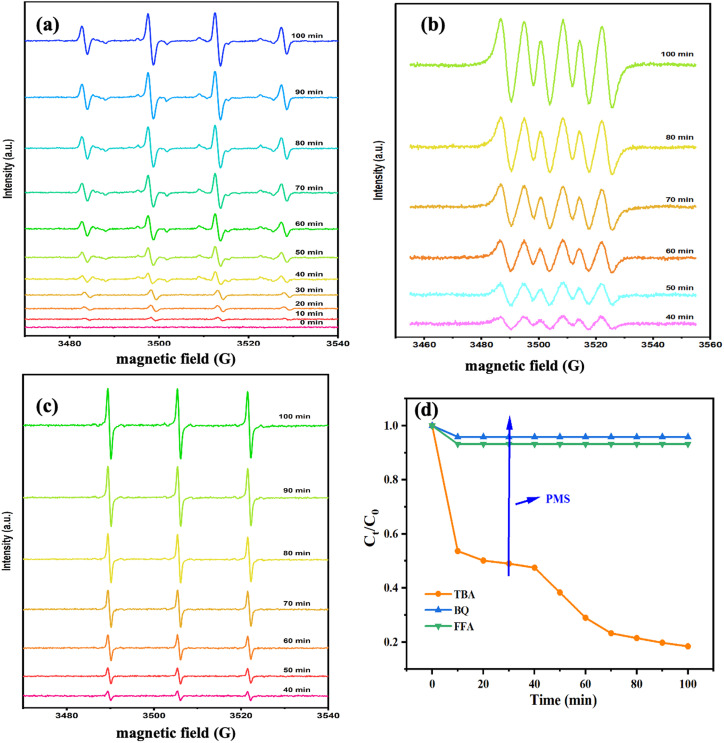
EPR spectra of Fe@Mn–BC for (a) DMP-˙OH, (b) DMPO-˙O_2_^−^, and (c) TEMP-^1^O_2_, and (d) quenching agents on CZB degradation.

To determine the impact of active species on CBZ degradation, EPR spectroscopy was employed to discern the ROS during the catalytic process. The free-radical spin traps were DMPO and TEMP. As seen in Fig. 5a, a typical pattern with an intensity of 1 : 2 : 2 : 1 was found. With increasing time, the intensity of DMPO-˙OH adducts increased gradually and reached a maximum at 100 min, indicating the continuous generation of ˙OH in the system. The typical signals for DMPO-˙O_2_^−^ are presented in Fig. 5b. The intensity of DMPO-˙O_2_^−^ adducts increased gradually from 40 min and reached the highest at 100 min. ˙O_2_^−^ did not appear before 40 min in this system. This was because PMS was added after 30 min of the reaction, and then ˙O_2_^−^ was generated subsequently, which implied that PMS promoted ˙O_2_^−^ production. The signal of TEMP-^1^O_2_ with a peak intensity ratio of 1 : 1 : 1 is illustrated in Fig. 5c. The intensity of TEMP-^1^O_2_ increased after 40 min in the catalytic process, suggesting that PMS was beneficial for ^1^O_2_ production. In summary, ˙OH was produced mainly by the Fe@Mn–BC catalyst during the first 30 min of the reaction. The addition of PMS induces the production of ˙O_2_^−^ and ^1^O_2_, and guided the CBZ degradation effectively. In addition, a quenching spectrum was conducted by different scavengers. BQ, FFA and TBA were employed as the scavenger for ˙O_2_^−^, ^1^O_2_ and ˙OH, respectively.^47,48^ As shown in Fig. 5d, CBZ degradation was not suppressed when TBA was added, indicating that ˙OH did not play an important part in CBZ degradation. However, in the presence of BQ and FFA, the degradation rates of CBZ were inhibited, which suggested that ˙O_2_^−^ and ^1^O_2_ had primary roles in CBZ degradation.

### 3.3 Activation properties of Fe@Mn–BC for PMS

**Fig. 6:**
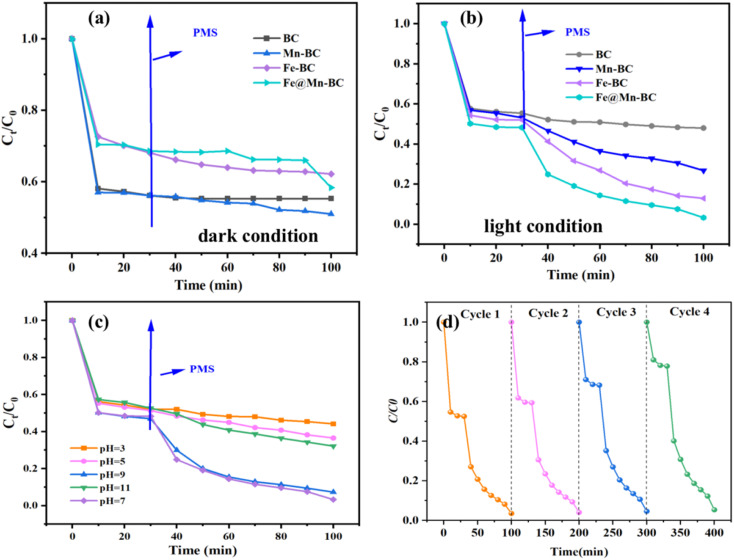
Degradation rate of different BC samples (a) under a dark condition and (b) under a light condition. (c) Degradation rate of Fe@Mn–BC under different pH values. (d) Stability of Fe@Mn–BC.

The activation properties of PMS were evaluated for all samples. PMS was added after 30 min in all reaction systems. The degradation properties of CBZ of different samples for PMS under a dark reaction condition are presented in Fig. 6a. The removal efficiencies of all samples were low at 30 min. Meanwhile, the degradation rates of CBZ did not improve significantly after PMS addition, which indicated that the catalysts had little effect on PMS activation. As shown in Fig. 6b, the removal efficiencies of CBZ were low. However, the degradation rate of CBZ by Fe@Mn–BC was 99% at 100 min when the PMS was in the system. The PMS/Fe@Mn–BC system had a higher degradation rate for CBZ under light, indicating that Fe@Mn–BC and light could stimulate PMS to degrade CBZ.

**Fig. 7:**
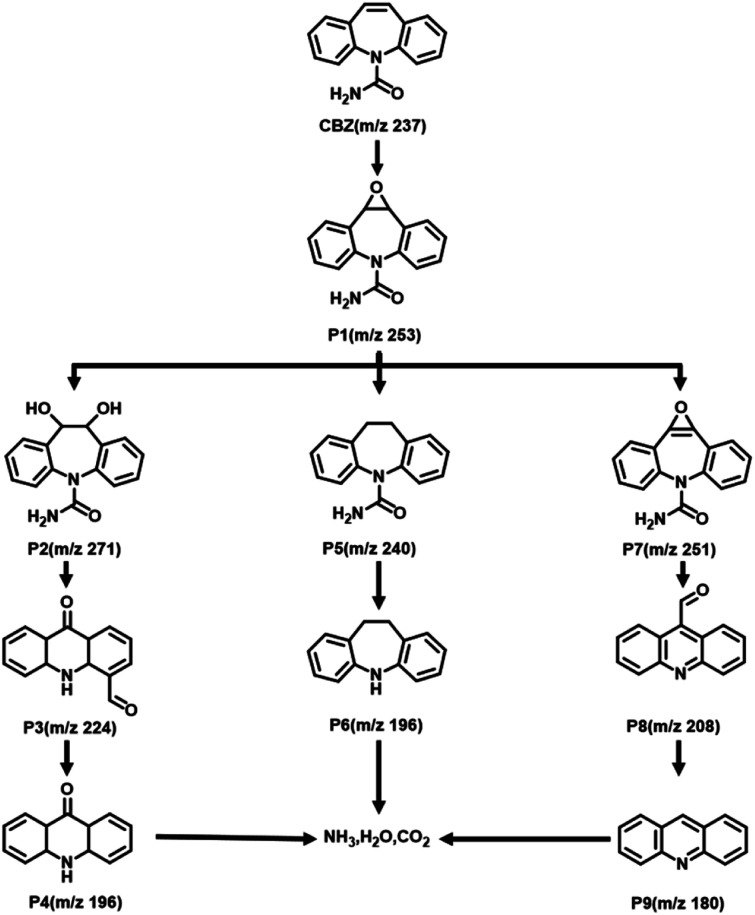
Possible degradation pathways of CBZ in the Fe@Mn–BC/PMS system.

The impact of the initial pH on CBZ degradation is displayed in Fig. 6c. The degradation performance was optimal (99%) under neutral conditions (pH = 7). The degradation rates of CBZ were relatively low when the initial pH was acidic. The degradation rates were 32% and 38% for pH = 3 and 5, respectively. The degradation rates of Fe@Mn–BC on CBZ were 91% and 63% when the pH was 9 and 11, respectively. Hence, Fe@Mn–BC had better reaction properties under weak alkali and neutral conditions. Moreover, the catalytic performances of different BC samples for PMS activation on the degradation of pollutants reported in other papers were compared (Table 1). The Fe@Mn–BC catalyst for PMS activation in this work showed an efficient photocatalytic performance for CBZ degradation. The structure of CBZ and degradation products are shown in Fig. 7. To evaluate the stability of Fe@Mn–BC, repeated catalytic tests were conducted by four degradation cycles of CBZ. According to Fig. 6d, the degradation efficiencies after four cycles were >95%. Taken together, our data suggest that Fe@Mn–BC could be a photocatalyst for CBZ removal.

**Table 1:** Comparative performance of BC materials for PMS activation on pollutant degradation

Catalyst	Degradation time (min)	Performance (efficiency (%))	Reference
Fe@Mn–BC	100 min	99%	This work
Fe–Cu bimetal–BC	90 min	90%	49
NOSB/PMS	40 min	67%	50
NBC–Fe–Cu	60 min	91%	51
PMS/BOSBC	60 min	98%	52
BC900/PMS	120 min	99%	53

## 4. Conclusions

Fe@Mn–BC catalysts were prepared by a hydrothermal method. Fe@Mn–BC was employed to activate PMS to degrade CBZ. Experiments based on free-radical detection and scavengers indicated that ˙O_2_^−^ and ^1^O_2_ had dominant roles in this degradation system. The catalyst Fe@Mn–BC exhibited superior catalytic activity for PMS activation under light radiation. When the initial pH was 7, the degradation rate of CBZ by Fe@Mn–BC was 99% at 100 min under light radiation. Degradation was more effective under light and a PMS-activated catalytic system. Hence, a promising BC-based catalyst for CBZ degradation was described.

## Conflicts of interest

The authors declare no conflicts of interest.

## Supplementary Material
